# ARK5 enhances cell survival associated with mitochondrial morphological dynamics from fusion to fission in human multiple myeloma cells

**DOI:** 10.1038/s41420-024-01814-w

**Published:** 2024-01-29

**Authors:** Sivasundaram Karnan, Ichiro Hanamura, Akinobu Ota, Lam Quang Vu, Kaori Uchino, Tomohiro Horio, Satsuki Murakami, Shohei Mizuno, Md Lutfur Rahman, Md Wahiduzzaman, Muhammad Nazmul Hasan, Mrityunjoy Biswas, Toshinori Hyodo, Hideaki Ito, Atsushi Suzuki, Hiroyuki Konishi, Shinobu Tsuzuki, Yoshitaka Hosokawa, Akiyoshi Takami

**Affiliations:** 1 Department of Biochemistry, Aichi Medical University, Nagakute, Aichi Japan; 2 Division of Hematology, Department of Internal Medicine, Aichi Medical University, Nagakute, Aichi Japan; 3 Department of Nutritional Environment, College of Human Life and Environment, Kinjo Gakuin University, Nagoya, 463-8521 Japan; 4 EuGEF Research Foundation, Chattogram, Bangladesh; 5 Department of Biochemistry, Emory University School of Medicine, Atlanta, GA USA; 6 Department of Foundations of Medicine, NYU Grossman Long Island School of Medicine, 101 Mineola Blvd, Mineola, NY 11501 USA; 7 Department of Pathology, Aichi Medical University, Nagakute, Aichi Japan; 8 Hematology Medical Franchise, Department of Medical Affairs, Novartis Japan, Tokyo, Japan

**Keywords:** Myeloma, Mechanisms of disease

## Abstract

5′ adenosine monophosphate–activated protein kinase–related kinase 5 (ARK5) is involved in mitochondrial ATP production and associated with poor prognosis of multiple myeloma (MM). However, the molecular mechanisms of ARK5 in MM remain largely unknown. This study examined the pathogenic role of ARK5 in mitochondria by using genetically modified isogenic cell clones with or without *ARK5* in human myeloma cell lines, KMS-11 and Sachi, which overexpress *ARK5*. The biallelic knockout of *ARK5* (ARK5-KO) inhibited cell proliferation, colony formation, and migration with increased apoptosis. Mitochondrial fusion was enhanced in ARK5-KO cells, unlike in *ARK5* wild-type (ARK5-WT) cells, which exhibited increased mitochondrial fission. Furthermore, ARK5-KO cells demonstrated a lower phosphorylated dynamin–related protein 1 at serine 616, higher protein expression of mitofusin-1 (MFN1) and MFN2, optic atrophy 1 with a lower level of ATP, and higher levels of lactate and reactive oxygen species than ARK5-WT cells. Our findings suggest that ARK5-enhanced myeloma cells can survive associated mitochondrial fission and activity. This study first revealed the relationship between ARK5 and mitochondrial morphological dynamics. Thus, our outcomes show novel aspects of mitochondrial biology of ARK5, which can afford a more advanced treatment approach for unfavorable MM expressing ARK5.

## Introduction

Multiple myeloma (MM) is a tumor arising from plasma cells that is generally incurable [1]. Elucidating the molecular mechanisms associated with poor prognosis can help develop effective target therapeutics [2]. In MM, the overexpression of large *MAF* (v-maf avian musculoaponeurotic fibrosarcoma oncogene homolog proto-oncogene) genes, including *MAF, MAFB*, and *MAFA*, characterized by chromosomal translocations into *immunoglobulin heavy chain* (*IGH*) loci [3–6], have been associated with poor prognosis [7, 8]. Large MAFs are DNA-binding, leucine zipper-containing transcription factors. 5′ adenosine monophosphate (AMP)-activated protein kinase–related kinase 5 (ARK5) is a known transcriptional target of large MAFs in MM [9] and a potential therapeutic target for improving outcomes in patients with MM. ARK5 (NUAK1, NUAK family, SNF1-like kinase 1) is a tumor survival factor that is Akt-promoted during nutrient starvation and serves as an ATM kinase [10]. ARK5 has been proved to be involved in the processes of tumor invasion and metastasis in colorectal, pancreatic, gastric, and liver cancers [10, 11]. In MM, ARK5 is upregulated by large MAFs connecting with MAF recognition element (MARE) in the part of downstream promoter genes, thus enhancing cell migration under IGF-1 [9]. ONO123300, a dual ARK5/CDK4 inhibitor, demonstrated antimyeloma effects against human MM cells overexpressing ARK5 in vitro [12]. However, apart from ONO123300, no ARK5-targeted drug candidate has been reported in MM. Recently, Escalona et al. demonstrated that ARK5 enhanced mitochondrial ATP production in several cancer cells, including breast, colon, and cervical cancers [13], indicating that ARK5 is involved in mitochondrial activity in cancer.

Mitochondria are central to intracellular energy production, including ATP, through oxidative phosphorylation and lipid oxidation [14]. They are pleomorphic and can adopt diverse structures depending on various cell characteristics [15]. Maintaining mitochondrial integrity and homeostasis is vital for optimal cellular function that is achieved by continual fusion and fission. An uneven balance in fission and fusion processes can result in either a fragmented or an overly fused group of mitochondria. Changes in mitochondrial function and morphology have been linked to the diseases, such as cancers and neurodegenerative disorders [14, 16–18]. Cell proliferation, invasion, and migration require increased oxidative phosphorylation (OxPhos), which is provided by mitochondrial fusion [19, 20]. Mitochondrial homeostasis between fission and fusion is also crucial to achieve a balance of cellular glycolysis [21], ATP levels, reactive oxygen species (ROS) levels, lactate production, cell cycle progression, and apoptosis [22]. In MM, the mitochondrial function has been proved to indicate on advanced stage of disease and drug resistance [23, 24]; however, the effects of ARK5 on mitochondrial biology are largely unknown. This study explored the pathogenic roles of ARK5, especially mitochondrial morphology, using biallelically ARK5-disrupted human myeloma cells and showed that ARK5 regulated morphological changes in mitochondria. Furthermore, as far as we know, our research is novel to investigate direct link between ARK5 and mitochondrial morphology.

## Results

### Expression of *ARK5* is associated with that of MAF or MAFB in human myeloma cell lines

**Fig. 1:**
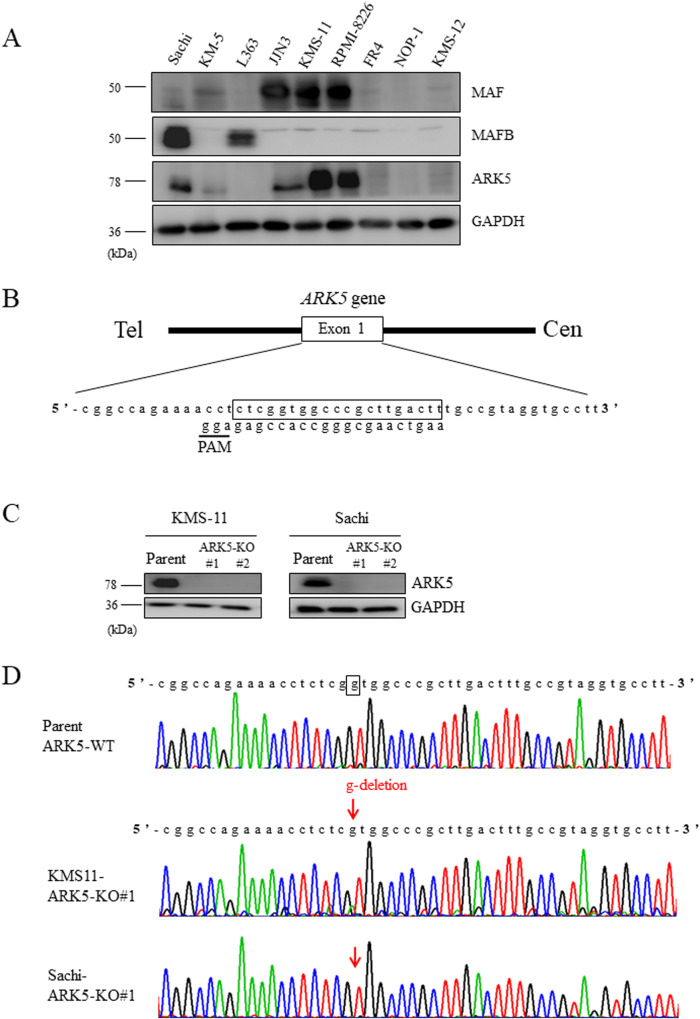
Generation of *ARK5* knockout (ARK5‐KO) clonal cells with CRISPR/Cas9 system involving human myeloma cell lines, KMS-11, and Sachi. **A** Western blot analysis for MAF, MAFB, and ARK5 expression in the MM cell lines. GAPDH was used as an internal control. **B** A single guide RNA sequence was developed against exon 1 of *ARK5* locus. **C** ARK5 protein expression was revealed via western blot analysis. **D** The genomic sequence analysis of *ARK5* in KMS-11 and Sachi-KO clonal cells was compared with that of parent cells.

The protein expression of MAF, MAFB, and ARK5 was analyzed in nine human myeloma cell lines: Sachi, KM5, L363, JJN3, KMS-11, RPMI-8226, FR4, NOP-1, and KMS-12. ARK5 expression was found in five cell lines (Sachi, KM5, JJN3, KMS-11, and RPMI-8226), revealing the presence of MAFB or MAF proteins, except for KM5 (Fig. 1A). There was no ARK5 expression in the cell lines having that for MAFB or MAF. The mRNA of *MAFB* was weakly expressed in KM5 [4], which has t(5;20); therefore, the protein levels of MAFB in KM5 may be too weak to be detected via western blotting. As reported previously [9], these results suggest that MAF or MAFB transcriptionally upregulates ARK5 expression in MM cells.

### *ARK5* knockout inhibits cell proliferation, colony formation, and cell migration in myeloma cells

**Fig. 2:**
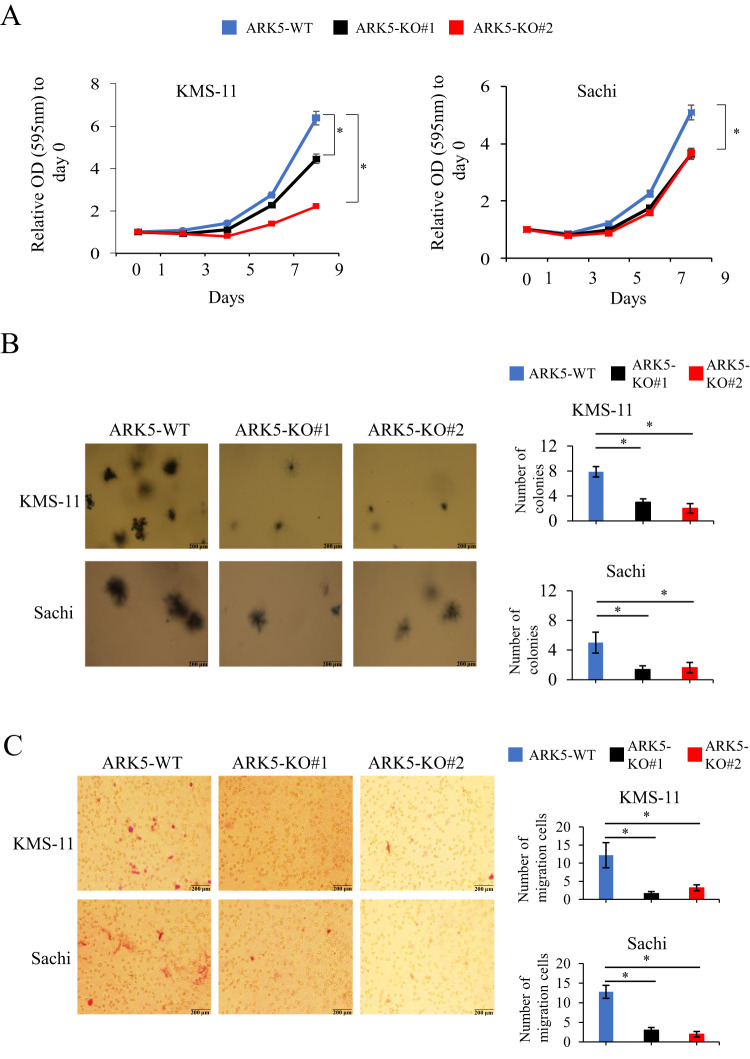
Cell growth and migration of ARK5-KO clonal and parental cells. **A** MTT analyses of the cultivation rate of parental and ARK5-KO clonal cells. The data were shown as the mean ± standard error of the mean (SEM) (*n* = 3). *P < 0.05 reflects a statistically significant difference between parental and KO cells. **B** Representative soft agar colony formation assays were obtained. The right bar graphs demonstrate the number of stained colonies. Scale bar = 200 μm. The data were presented as the mean ± SEM (*n* = 3). *P < 0.05 was considered a statistically significant difference. **C** Representative migration assays using the Boyden chamber are received. The right bar graph shows the number of stained colonies. Scale bar = 200 μm. The data were outlined as the mean ± SEM (*n* = 3). Asterisks (*) point on statistically significant differences (*P < 0.05).

To understand the role of ARK5 in myeloma cells, we established *ARK5*-knockout (ARK5-KO) cell clones using CRISPR-Cas9 system that targeted a sequence in exon 1 of *ARK5* in the Sachi and KMS-11 cells (Fig. 1B, C). Sequence analysis confirmed the bi-allelic deletion of G in the Sachi and KMS-11 cell lines (Fig. 1D). Using these isogenic cell clones with or without *ARK5*, we examined cell proliferation using an MTT assay. The cell growth of ARK5-KO cells was significantly diminished compared to that of parental ARK5-WT cells (Fig. 2A). The colony formation and migration abilities of ARK5-KO cell clones were lower than those of parental ARK5-WT cells (Fig. 2B, C), indicating that ARK5 expression is essential for cell growth in MAF or MAFB-expressing MM cells.

### *ARK5* knockout increased apoptotic cells and the cell cycle S-phase ratio in myeloma cells

**Fig. 3:**
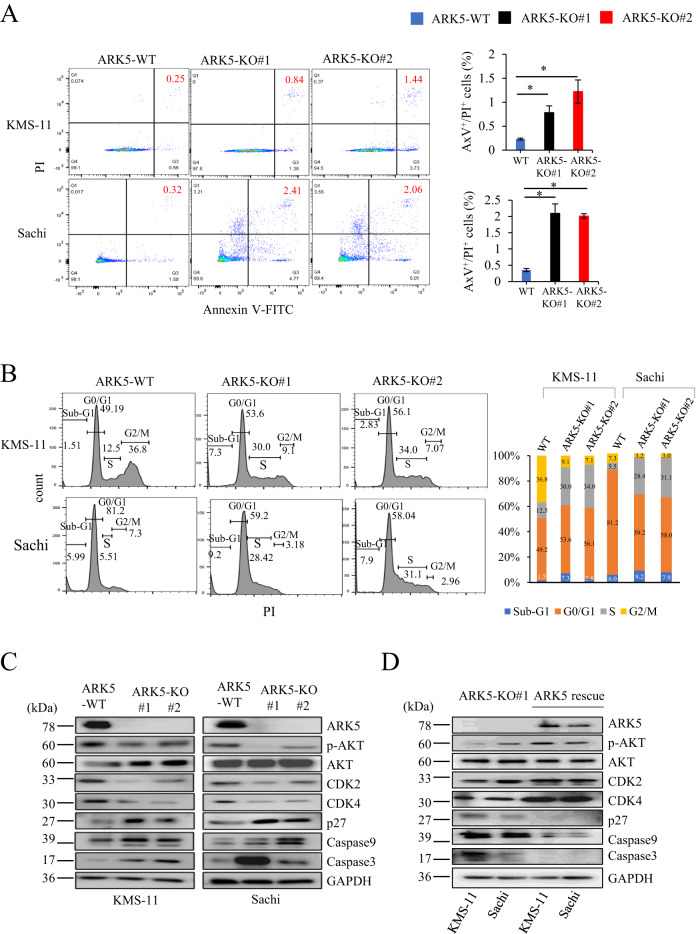
Effects of ARK-5-KO on apoptosis and cell cycle in myeloma cells. **A** Apoptosis assay via annexin V-based staining method. The proportion of apoptotic cells was higher in ARK5-KO cells than in ARK5-WT cells. The right bar graphs represented the proportion of apoptotic (Annexin V^+^/PI^+^) cells. The data were presented as the mean ± SEM (*n* = 3). Asterisks (*) show statistically significant differences (*P < 0.05). **B** Cell cycle was analyzed via LSRFortessa X-20 Flow Cytometer and FlowJo software. S-phase ratio was elevated in ARK5-KO cells compared to their parental cells. **C** Western blot analysis reflecting protein expression of ARK5, phosphorylation levels of AKT, cyclin-dependent kinase (CDK)2, CDK4, p27, caspase 9, and caspase 3 in parental (KMS-11 and Sachi), KMS-11-ARK5-KO and Sachi-ARK5-KO cells. **D** Effect of exogenous ARK5 expression in ARK5/ARK5-KO cells (rescue) on the protein levels of ARK5, phosphorylation levels of AKT, CDK2, CDK4, p27, caspase 9, and caspase3 compared with ARK5-KO cells in KMS-11 and Sachi.

Because ARK5-KO cell clones exhibited decreased cell proliferation compared to parental ARK5-WT cells, we performed an apoptosis assay in ARK5-KO and ARK5-WT cells (Fig. 3A). The proportion of apoptotic cells was higher in the ARK5-KO cell clones compared to that in the parent cells. Additionally, there was an increased proportion of S-phase in that ARK5-KO clone cells than in the parent cells (Fig. 3B). The expression levels of phosphorylated AKT, caspase3 and caspase9, and p27 were increased in ARK5-KO cells, while CDK2 and CDK4 expression was decreased (Fig. 3C), suggesting that *ARK5* knockout promotes apoptosis and cell cycle arrest in myeloma cells. To confirm the impact of ARK5 on the expression of these proteins, an exogenous ARK5/ARK5-KO#1 cell clone was employed in this study. Rescuing the ARK5 protein expression resulted in the alteration of protein expression and phosphorylation, which were all nullified in the exogenous ARK5/ARK5-KO cell clones as compared to that observed in the ARK5-KO cell clones (Fig. 3D).

### *ARK5* knockout affects mitochondrial morphology and activity in myeloma cells

**Fig. 4:**
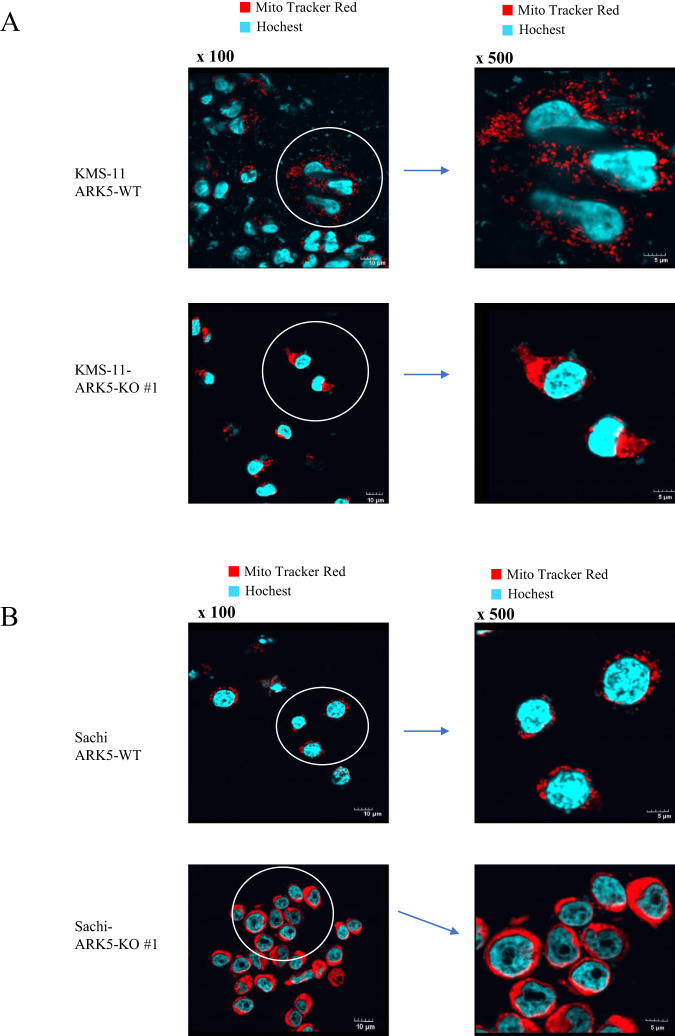
Effects of ARK-5-KO on mitochondrial morphology in myeloma cells. **A**, **B** Staining of mitochondria and nuclei was done with MitoTracker Red and Hoechst (blue), respectively, in human myeloma cells. ARK5-KO cells exhibited mitochondrial fusion, while ARK5-wild type (ARK5-WT) cells showed mitochondrial fission. ARK5-WT showed a spotty distribution of mitochondria (upper panel). Conversely, ARK5-KO cells demonstrated mitochondrial fusion (lower panel) in both myeloma cell lines, KMS-11 and Sachi. The right panel shows the magnification of the left panel.

**Fig. 5:**
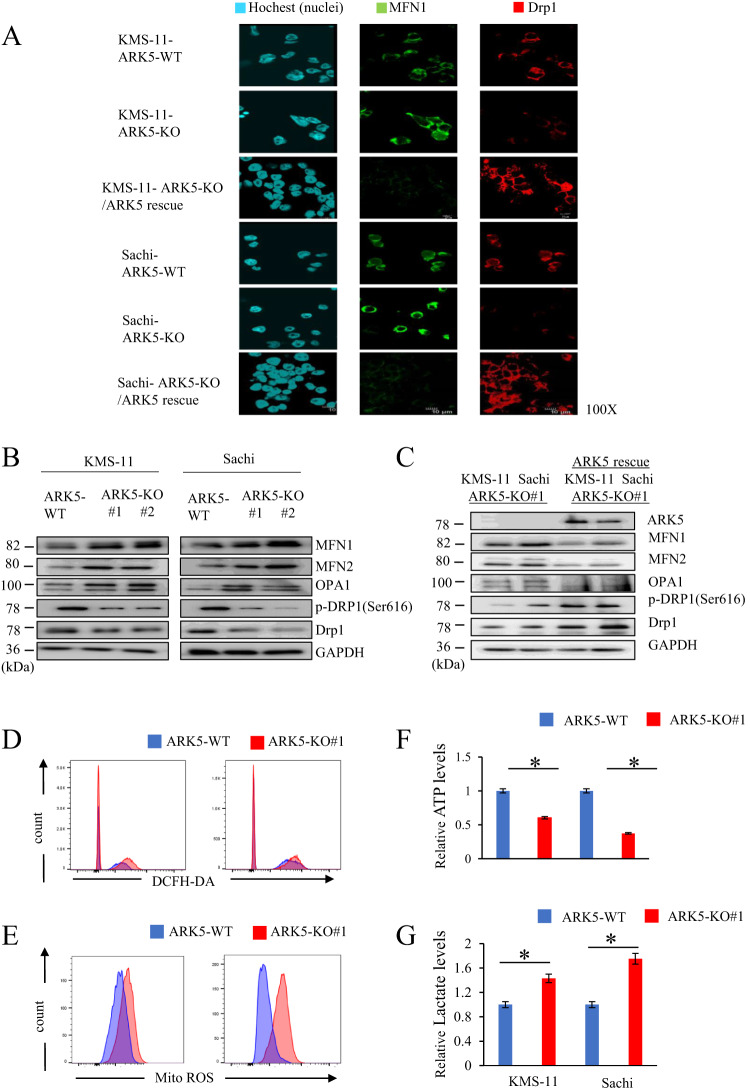
Effects of ARK-5-KO on the expression of GTPase associated with mitochondrial morphology in myeloma cells. **A** Mitofusin-1 (MFN1) (green) and dynamin-related protein 1 (DRP1) (red) were co-stained using Hoechst (blue) in human myeloma cells. MFN1 was increased in ARK5-KO cells compared to that in ARK5-WT cells and ARK5/ARK5-KO cells. DRP1 decreased in ARK5-KO cells compared to that in ARK5-WT cells and ARK5/ARK5-KO cells. **B**, **C** Western blot analysis of MFN1, MFN2, optic atrophy 1 (OPA1), phosphorylated DRP1 at serine 616, and DRP1 in parental and ARK5‐KO clone cells and ARK5/ ARK5-KO cells. **D**, **E** Reactive oxygen species (ROS) production was measured via dichloro-dihydro-fluorescein diacetate assay and mitochondria ROS assay in parental and ARK5‐KO clone cells. **F** Adenosine triphosphate (ATP) production was evaluated using ATPlite Luminescence assay. The bar graphs present ATP levels relative to the parent ARK5-WT cells. **G** Relative lactate levels to the parent ARK5-WT cells. The data were shown as the mean ± SEM (*n* = 3). Asterisks (*) indicate statistically significant differences (*P < 0.05).

To evaluate the role of ARK5 in mitochondrial function, we first analyzed the mitochondrial morphology. The ARK5-KO cells displayed elongated mitochondrial structure, indicating an elevation of mitochondrial fusion over fission. Contrarily, the ARK5-WT cells exhibited a fragmented mitochondrial structure, depicting the opposing function of elevated fission over fusion (Fig. 4A, B). The preserved and unaltered cellular morphology in both the ARK5-WT and ARK-KO cells indicated that the mitochondrial structural changes are not a result of cell death or cell shrinkage caused by ARK5-KO (Supplementary Fig. 5), suggesting that *ARK5* knockout enhances shifting of mitochondrial fusion from fission. We next assessed the expression level of proteins associated with mitochondrial morphology, including mitofusin-1 (MFN1), mitofusin-2, (MFN2), optic atrophy-1 (OPA1), and dynamin-related protein 1 (DRP1). ARK5-KO cells demonstrated a lower phosphorylated DRP1 at serine 616, decreased expression of DRP1, and elevated expressions of MFN1, MFN2, and OPA1 compared to parent cells (Fig. 5A, B). The immunofluorescence data showed that the exogenous expression of ARK5 in the ARK5-KO clones decreased the MFN1 expression and increased the Drp1 expression (Fig. 5A). Furthermore, these increases and decreases were all revoked by the exogenous expression of ARK5 in the ARK5-KO clone (Fig. 5C). These results suggest that ARK5 is involved in mitochondrial dynamics. We next assessed levels of intracellular reactive oxygen species (Total ROS), Mitochondrial ROS (Mito ROS), ATP, and lactate in the *ARK5*-KO and parent cells. *ARK5*-KO cells showed higher levels of ROS, and lactate and a lower level of ATP (Fig. 5D–G).

### *ARK5* depletion significantly inhibits growth of KMS-11 in xenografted mice

We assessed the in vivo effect of *ARK5*-KO on the growth of KMS-11cells using xenografted mice. Our findings demonstrated that the *ARK5* knockout significantly suppressed the tumor growth as compared to the *ARK5*-WT control (parent) in vivo (Supplementary Fig. S1A,B) without causing weight loss (Supplementary Fig. S1C).

### Effects of apoptosis inhibitors on cell proliferation in ARK5-WT cells

**Fig. 6:**
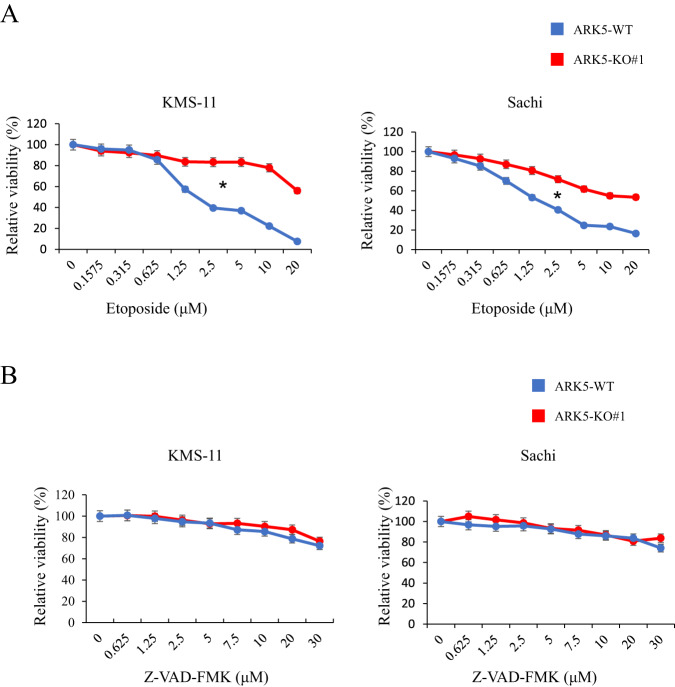
Effect of the apoptosis inhibitors on ARK5-WT and ARK5-KO cell proliferation. **A** Effect of etoposide and **B** Z-VAD-FMK on KMS-11, KMS-11-ARK5-KO#1, Sachi, and Sachi–ARK5-KO#1 cell proliferation. Cells (1 × 10^3^ cells/well) were subjected to treatment with the mentioned concentration of inhibitors for 48 h. Cell growth was estimated by performing MTT assay. Blue and red lines indicate KMS-11, Sachi and KMS-11-ARK5-KO#1, Sachi–ARK5-KO#1 cells, respectively. Data are shown as mean ± SE (*n* = 3). Asterisks (*) reflect statistically significant differences (*P < 0.05).

To explore the effects of apoptosis inhibitors on cell proliferation in both the ARK5-WT and ARK5-KO cells and to gain a deeper understanding of their efficacy in KMS-11-ARK5-WT and Sachi-ARK5-WT cells, we conducted MTT assays. These assays allowed us to assess the growth of ARK5-WT and ARK5-KO cells under the influence of apoptosis inhibitors, etoposide, and a caspase inhibitor, Z-VAD-FMK. We found that the ARK5-WT cells exhibited higher sensitivity to etoposide as compared to their counterparts, KMS-11-ARK5-KO#1 and Sachi-ARK5-KO#1 cells (Fig. 6A). Conversely, using the Z-VAD-FMK inhibitor did not produce a significant alteration in the survival rates of either the parental or ARK5-KO cells (Fig. 6B). These findings imply that etoposide may offer potential benefits in clinical applications for the treatment of MM patients with ARK5 overexpression.

## Discussion

This study demonstrated that ARK5 enhanced cell growth and migration of MM cells that was shown in the application of human myeloma isogenic cell clones with or without *ARK5*. *ARK5* knockout causes the rightward shift from fission to fusion, indicating that MM cells have elevated mitochondrial fission, which represent the association of ARK5 with mitochondrial morphological dynamics in MM cells. The ARK5-KO cells showed decreased phosphorylated DRP1 levels, increased expression of MFN1, MFN2, and OPA1, and decreased ATP levels; increased lactate and ROS levels compared to parental ARK5-WT cells. Thus, our findings indicate that ARK5 helps myeloma cells survive by enhancing mitochondrial function associated with mitochondrial fission.

In MM, ARK5 expression is upregulated in cells overexpressing MAF or MAFB, mediated by IGH translocations. In this study, ARK5 was expressed in MAFB-overexpressing Sachi, MAF-overexpressing JJN3, KMS-11, and RPMI-8226, but L363 lacked ARK5 expression despite MAFB overexpression. L363 might have mutations in the MARE region, to which large MAFs bind. KM5 had t(5;20) translocation and expressed MAFB mRNA, but the MAFB protein remained undetected. Our results are consistent with those from a previous study stating that ARK5 expression was transcriptionally upregulated by large MAFs [9]. Higher ARK5 expression was linked to overall unfavorable survival (*p* = 0.032) in 290 patients with newly diagnosed MM according to data extracted from a public data domain (GSE19784, HOVON-65/GMMG-HD4) [25, 26] (Supplementary Fig. S2). Additionally, we measured the ARK5, MAF, and MAFB expressions at the RNA levels by performing an EZR analysis with the abovementioned public data. We obtained a positive relationship between ARK5 and MAF (Supplementary Fig. 4A) and between ARK5 and MAFB (Supplementary Fig. S4B). To investigate the molecular functions of ARK5 in MM, we established a cellular model of ARK5-KO by CRISPR-Cas9 system using human MM cell lines, KMS-11 and Sachi. ARK5-KO proved to inhibit cell proliferation, colony formation, and cell migration compared to ARK5-WT cells (Fig. 2A, B, C) with significantly increased proportion of apoptotic and S-phase cells (Fig. 3A, B), suggesting that ARK5-KO induced apoptosis and cell cycle arrest. Additionally, we found that ARK5 altered the morphology of mitochondria from fusion to fission and increased ATP levels. (Fig. 5A, B, F).ARK5-KO cells exhibited higher levels of ROS and lactate than their parental ARK5-WT cells.

Mitochondrial fusion promotes cell proliferation by enhancing OxPhos [19], but elevated OxPhos and lactate levels don’t always indicate increased cellular ATP levels. The changes in ATP consumption or production could contribute to altered cellular ATP levels, which also depends on a specific cell type [27, 28]. Under hypoxic conditions, OxPhos fails to generate ATP, leading to an ATP deficiency and increased ROS production in the ARK5-KO cells [29]. Cytosolic ARK5 reportedly increases the ATP level [13], and inhibiting the ARK5 activity may cause a decline in the ATP levels in cells with an abnormal MYC expression, initiating diverse pro-apoptotic responses [30]. Consistent with our cell cycle arrest at S-phase (Fig. 3B) and the presence of hyperfused giant mitochondria (Fig. 4A, B) in ARK5-KO, the cells provide strong evidence on the involvement of ARK5 in mitochondrial fission, showing a scenario where ARK5-KO cells exhibit vice versa.

Mitochondria are morphologically dynamic and regulated by a balance between fusion and fission [31, 32]. GTPases, such as DRP1, MFN1, MFN2, and OPA1, control mitochondrial morphological dynamics. DRP1 is activated via phosphorylation by multiple kinases, including RAS [33–35], and is involved in mitochondrial fission. MFN 1, MFN2 and OPA1 mediate mitochondrial fusion [36]. We performed a kinase assay to explore the interaction between ARK5 and its substrates, including DRP1, MFN1, MFN2, and OPA1. However, we did not observe direct interactions between ARK5 and DRP1, MFN1, MFN2, or OPA1 (Supplementary Fig. S3). Based on our results, ARK5 indirectly regulates DRP1 phosphorylation, possibly through the involvement of other ARK5 downstream kinases. Herein, ARK5-WT cells demonstrated mitochondrial fission and higher phosphorylation of DRP1 [37, 38] at serine 616 (DRP1^S616^), and lower expression levels of MFN1, MFN2, and OPA1 (Fig. 5B), similar to oncogenic RAS mutant–mediated phosphorylation of DRP1^S616^ and mitochondrial fission in several human cancers, such as melanoma, and breast cancers [39–41]. Hence, DRP1^S616^ phosphorylation can be a target for MM expressing ARK5. Moreover, our research has shown a substantial decrease in cell growth in ART5-WT, as compared to ARK5-KO upon the administration of the etoposide drug (Fig. 6A). To the best of our knowledge, etoposide, a prominent antitumor agent for various cancers [42], has not been investigated in any study, specifically in terms of its efficacy on ARK5-enhanced MM cells. This potential warrants further investigation in future studies.

In conclusion, our study revealed that ARK5 expression was closely associated with MAF or MAFB expression and that ARK5 knockout suppressed cell growth and migration in MM. ARK5 expression altered mitochondrial morphology from fusion to fission mediated via increased DRP1^S616^ phosphorylation, which may have been a good target for treating unfavorable patients with MM expressing ARK5.

## Materials and methods

### Cell culture

The cultures representing multiple myeloma that were Sachi, KM5, L363, JJN3, KMS-11, RPMI-8226, FR4, NOP-1, and KMS12, were cultured in RPMI-1640 with 10% fetal bovine serum at 37 °C in a 5% CO_2_ humidified environment. All cell lines did not require any cytokines, including IL-6.

### CRISPR/Cas9 system for ARK5 gene knockout

The CRISPR/Cas9 system was employed to discontinue the expression of *ARK5* genes according to the results of a previous study [43]. The sgRNA sequence of *ARK5* exon 1 was 5′-AAGTCAAGCGGGCCACCGAG-3′ (Fig. 1B). Using vector backbone px458, knockout clones was established and a single clone was selected, expanded, and applied for biological assays. For the evaluation of sequences and electrophoresis, the amplifications of *ARK5* gene were performed by PCR with the following primers: Fw, 5′-TCGCCCCGCG CTTGGACATG GAA-3′ and Rev, 5′-GGTGCTGGAGAAAGAGTGAG-3′. The further sequence analysis was carried out to confirm the deletion of 1 bp in two independent KMS-11 and Sachi clones (designated #1 and #2) (Fig. 1C, D). For the ARK5 rescue, an ARK5/ pQCXIP-GFP vector was transfected into ARK5-KO#1 clone with the 4D-Nucleofector System having the pQCXIP-GFP control vector. The cells were then cultivated for 48 h, rinsed with phosphate-buffered saline (PBS), and lysed in buffer solution. To check for ARK5 expression, the obtained lysates underwent western blot analysis.

### Cell growth assay

A cell growth assay was done, as shown above [44, 45]. Briefly, cells (1 × 10^3^ cells/well) were incubated on 96-well plates. The optical density (595 nm) of the colored product was determined at 0, 1, 3, 5, and 7 days with SpectraMAX M5 (Molecular Devices, Sunnyvale, CA, USA).

### Soft agar colony formation assay

Soft agar colony formation assay was conducted following the previously described protocol [46, 47]. The number of colonies was counted using Colony Counter (Keyence, Tokyo, Japan). The descriptive statistics included mean ± standard error (*n* = 3).

### Migration assay

The migration assay was carried out according to the procedures outlined in prior studies [47] Cells (2.5 × 10^5^ per well) were cultivated in Boyden chambers on 24-well plates. The descriptive statistics represented mean ± standard error (*n* = 3).

### Cell cycle analysis

Briefly, 5 × 10^5^ cells were supplemented to each well of a six-well tissue culture plate. The cells were gathered after 48 h and immersed in 70% ethanol for a night at −30 °C. Then, they were resuspended and incubated with RNase A (100 μg/mL) at 37 °C for 30 min, labeled with PI (100 μg/mL), and incubated for another 30 min at 4 °C. Finally, the PI labeled cells were analyzed by Flowcytometry (LSRFortessa X-20 Flow Cytometer, BD Biosciences).

### Annexin V assay

The cells were cultivated on six-well culture plates (5 × 10^5^ cells/well). On the following day, they were mixed with annexin V (Ax)-FITC and PI (10 μg/mL) at 25 °C and incubated for 15 min. In the end, fluorescence intensities were evaluated with LSRFortessa X-20 Flow Cytometer (BD Biosciences).

### Western blot analysis

Western blot analysis was carried out according to the protocols of previous studies [48, 49] Supplementary Table S1 reflects the antibodies used in the detection of immune complexes that was done with Immuno Star LD (Wako Pure Chemical Industries, Ltd., Osaka, Japan) and LAS-4000 (GE Healthcare, Tokyo, Japan).

### Immunofluorescence

The KMS-11 and Sachi and their ARK5-KO cells were cultivated on glass coverslips and immersed in 4% paraformaldehyde solution for 20 min at room temperature. Cells were saturated with PBS having 0.1% Triton X-100 and fixed using PBS with 7% serum for 30 min, while being targeted by primary antibodies. Then Alexa Fluor-conjugated secondary antibodies (Invitrogen) were added. To visualize mitochondria, cell staining done using Mitotracker (stock solution 1 mM; 1:10,000 dilution) for 1 h at 37 °C. Cells were rinsed with PBS and stored in cold paraformaldehyde (3.2% in PBS) for 20 min at room temperature. After washing, samples were mounted with PermaFluor, and subsequently, the images were acquired with FLUOVIEW FV3000 Series of Confocal Laser Scanning Microscopes.

### DCHF-DA-based DCF assay

The cell cultures were placed in six-well plates (5 × 10^5^ cells/ well). On the following day, 10 μM DCFH-DA (2′,7′-Dichlorodihydrofluorescein diacetate) was added in the medium and kept for 15 min in the dark. Then, DCFH-DA was taken for analysis, and cells were rinsed with PBS, isolated with trypsin, immersed in the medium, centrifuged for 5 min at 1000 rpm, plunged in PBS in 0.5-mL tubes, positioned on ice, and analyzed with FACS.

### MitoROS assay

The cells were grown in six-well culture plates (5 × 10^5^ cells/well). On the following day, they 10-μM mitoROS (AAT Bioquest Cat#: 16052) was added and incubated at 37 °C for 45 min. Eventually, the fluorescence parameters were estimated with LSRFortessa X-20 Flow Cytometer.

### ATP assay

The KMS-11 and Sachi and their ARK5-KO#1 cells were cultivated in a 96-well plate (2 × 10^4^ cells/well) for 12 h. ATPlite 1step Luminescence Assay System kit (PerkinElmer cat. #6016736) was applied for ATP analysis. In a short, 100 μL of the reconstituted substrate solution was added per well. After 10 min of growing at RT, the analysis of luminescence was done with Microplate Reader (MD SpectraMax M5). The received data were shown as mean ± standard error (*n* = 3).

### Lactic acid assay

The KMS-11 and Sachi and their ARK5-KO#1 cells were put in a 6-well plate (1 × 10^5^ cells/well) and stored for 24 h for the further analysis with lactate assay kit-WST (Dojindo cat. #L256). Briefly, after the cultivation, the sample solution was obtained by adding 20 μl of cell culture supernatant to a 1.5-ml microtube and 100 times-dilution with 0.1% Triton X-100, followed by the distribution of each 20 μl to all wells. Meanwhile, a working solution (80 μl) was placed in each well. The 96-well microplate was then kept at 37 °C for 30 min. The absorbance at 450 nm was determined with a microplate reader (SpectraMAX M5). Data were reflected as mean ± standard error (*n* = 3).

### In vitro kinase assay

In vitro kinase assay was done, as shown in the former studies [50]. Briefly, GST protein expressed in *Escherichia coli* was lysed with a RIPA buffer (50-mM Tris-HCl pH 7.4, 150-mM NaCl, 0.1% SDS, 0.5% DOC, 1% NP-40) having a complete protease inhibitor cocktail (Roche) and bound to Glutathione Sepharose 4B beads (GE Healthcare). Then, the GST protein was rinsed with a kinase buffer (40-mM Tris-HCl pH 7.4, 20-mM MgCl_2_, 0.1-mg/ml bovine serum albumin, and 50-μM dithiothreitol) and eluted using a kinase buffer containing 20-mM reduced glutathione. The RC-DC Protein Assay (Bio-Rad) was applied to measure the GST protein levels.

For the kinase assay, 16.7-ng/μl substrate candidate protein, 0.33-ng/μl ARK5 kinase, 33.3-nCi/μl (11 nM) [γ32-P] ATP, and 50-µM non-radiolabeled ATP were mixed in a kinase buffer (reaction volume of 30 μl) and stored for 30 min at 30 °C. The end of the reaction was marked by blending with 4× Laemmli sample buffer (40% glycerol, 270-mM Tris-HCl pH 6.8, 8% SDS, 20% 2-mercaptoethanol, 0.006% BPB). Then, the samples were detached by SDS-PAGE. In the end, the autoradiography image was obtained with BAS-5000 (GE Healthcare), Image Reader BAS-5000 Version 1.8 (Fujifilm), and Multi Gauge Version 3.1 (Fujifilm) software.

### Xenograft experiments

Animal experiment was performed, as detailed in previous studies [44] The use of animals in this study received the approval from the Ethics Committee of the Aichi Medical University Animal Experimentation Facility. All the protocols for the experiments involving mice in our research were done according to appropriate animal husbandry guidelines and regulations. Female nude mice (BALB/cSlc-nu/nu) (5 weeks old, each weighing 14 g) were obtained from CLEA Japan, Inc (Tokyo, Japan) and bred in pathogen-free conditions at the Institute of Animal Experiments in Aichi Medical University. For xenografting, 1 × 10^7^ KMS-11 and KMS-11-ARK5-KO#1cells were applied.

### Statistical analysis

The descriptive statistics was reflected as mean ± standard error. The outcomes were compared between the groups with two-tailed non-paired one-way analysis of variance with post hoc student’s *t-*test as required. A *p* value of <0.05 (indicated by asterisks) was regarded statistically significant. Statistical Package for the Social Sciences software was applied for the evaluation (version 23.0; SPSS, Inc., Chicago, IL, USA).

### Supplementary information

Supplementary information

## Data Availability

All the available data are presented either in the main manuscript or in the supplementary information. All the full and uncropped western blot data used in the study has been included in the supplementary information. Also, particular queries, data and materials are available based on reasonable request to corresponding author.
