# Supplementary material for: ARK5 enhances cell survival associated with mitochondrial morphological dynamics from fusion to fission in human multiple myeloma cells
Source: Cell Death Discov. 2024 Jan 29;10:56. doi: 10.1038/s41420-024-01814-w (PMC10822851; doi:10.1038/s41420-024-01814-w)
Supplement: Supplementary file 1 — Supplementary information [file 41420_2024_1814_MOESM1_ESM.pdf]

Supplementary information

Manuscript Title:

ARK5 enhances cell survival associated with mitochondrial morphological dynamics from fusion to fission in human multiple myeloma cells

Supplementary information includes 5 supplemental figures and 1 supplemental table.

All the full and uncropped version of the western blot images used in the manuscript has been included at the end of this file.

Supplemental Fig.S1

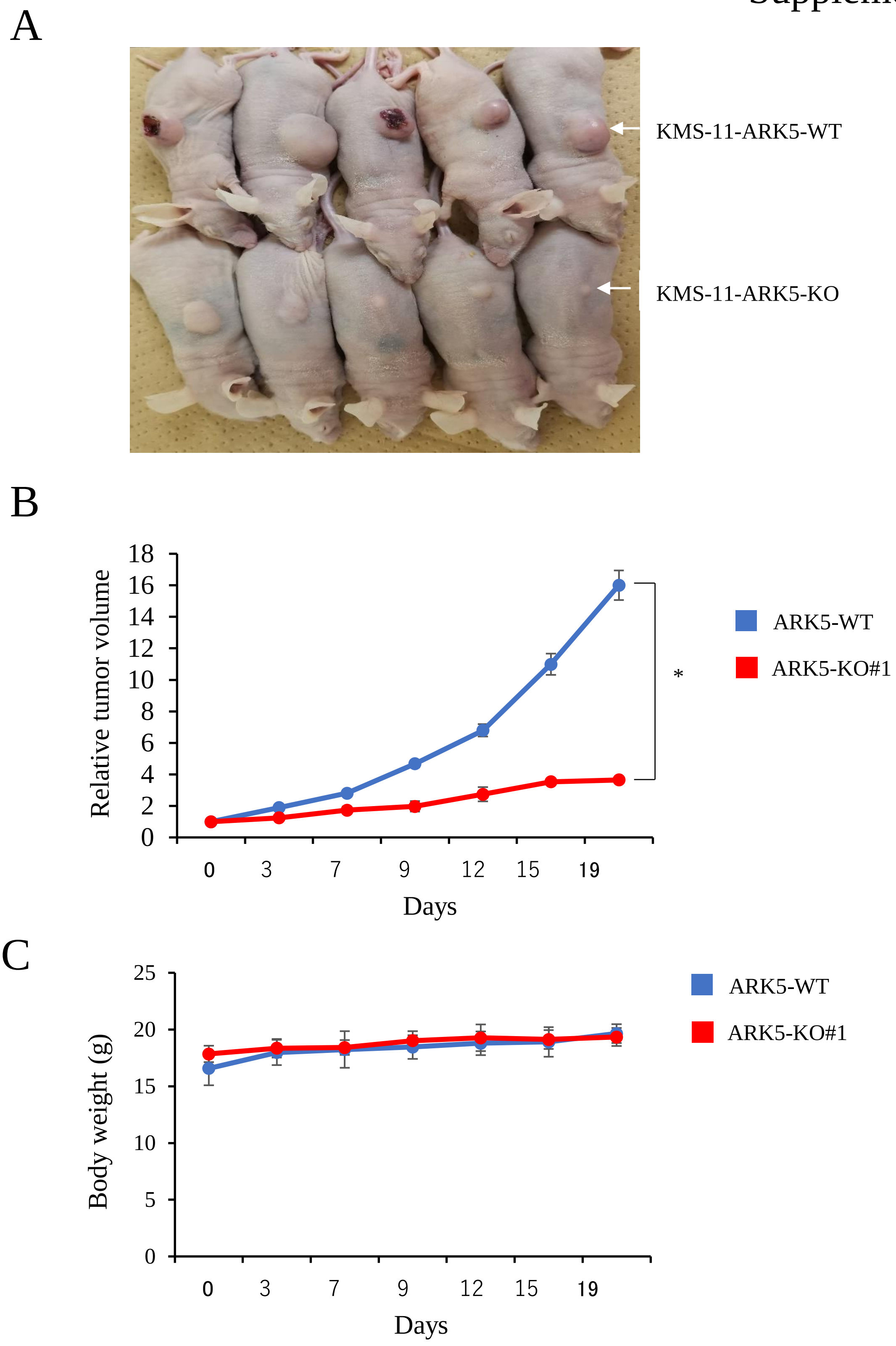

Figure legends S1: Effect of depletion on the tumor growth of KMS-11cells in vivo

The KMS-11 (ARK5$^{+/+}$; 1 × 10^7 cells/mouse) and KMS-11-ARK5-KO#1 (ARK5$^{-/-}$; 1 × 10^7 cells/mouse) cells were subcutaneously injected into nude mice. After the tumor volume attained 50 mm^3 at day 0, subsequent measurements were done at day 3, 7, 9, 12, 15 and 19 into xenografted mice. (**A**) A representative image of xenografted tumors in each group. (b and c) Line graphs of the relative tumor volume (**B**) and body weight (**C**) of mice. The tumor volume was shown relative to tumor size on day 0, arbitrarily defined as 1. Data are shown as mean ± SE (n = 5). Asterisks (*) indicate statistically significant differences (*$P < 0.05$)

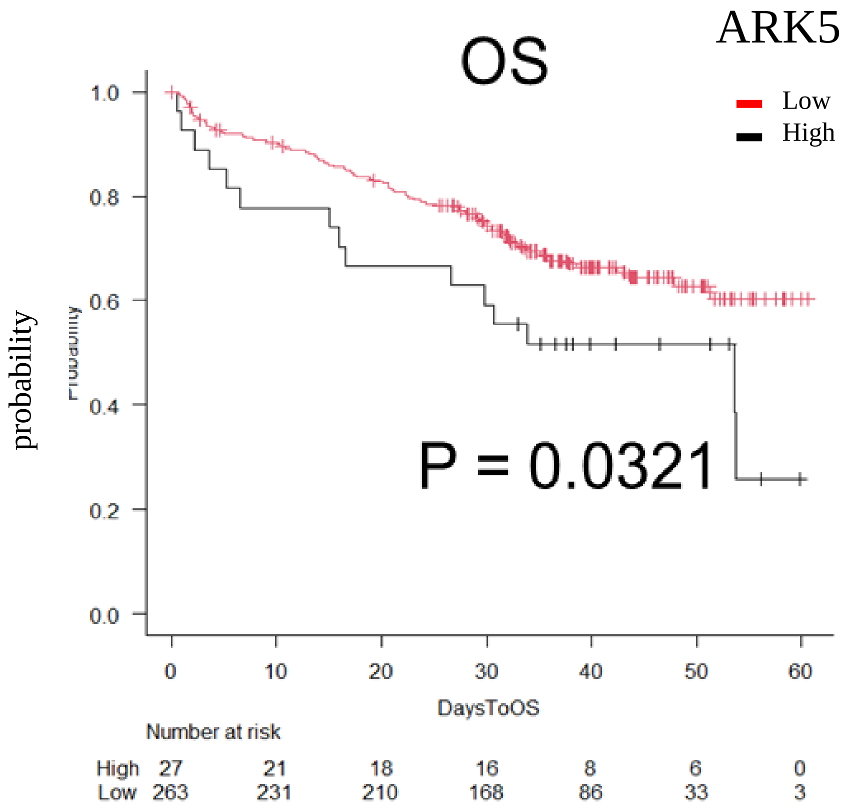

Figure legend S2: Relationship of MAF to ARK5 expression in patient samples.
Overall survival conforming to expression levels of ARK5 and analyzedvia Kaplan–Meier survival curve in 290 newly diagnosed with multiple myeloma and enrolled in the HOVON65/GMMG-HD4 trial patients. The data regarding survival and ARK5 expression were obtained from GSE19784.

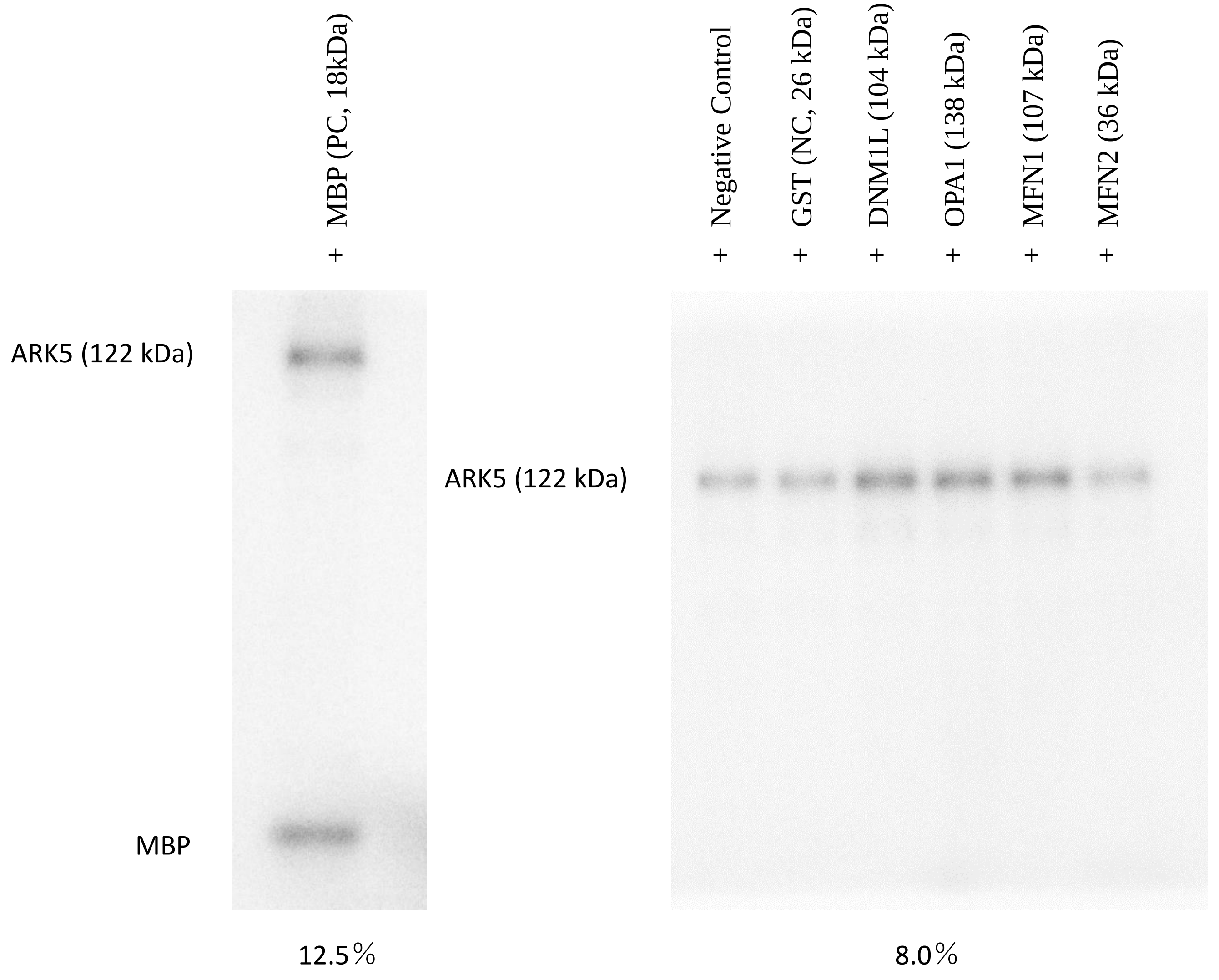

Figure legend S3: Effect of GST-DNM1L, GST-OPA1, GST-MFN1, and GST-MFN2 on ARK5 activity. Left panel: SDS-PAGE 12.5% gel using positive controls (Myelin Basic Protein, MBP). RRight panel: SDS-PAGE 8.0% gel using negative controls; GST (26 KDa) , GST-DNM1(104 KDa) L, GST-OPA1 (138 KDa), GST-MFN1 (107 KDa), and GST-MFN2 (36 KDa). The image was obtained using BAS-5000 (GE Healthcare) and Image Reader BAS-5000 Version 1.8 (Fujifilm), and analyzed with Multi Gauge Version 3.1 (Fujifilm) software.

A

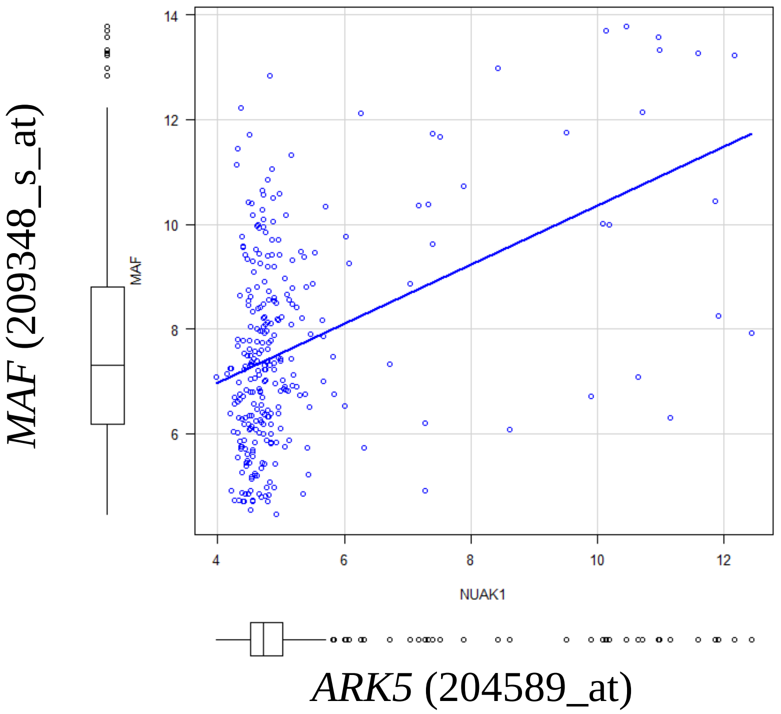

Spearman’s rank correlation coefficient (n = 290)

R = 0.324 P = 1.91 $\times$ 10^{-8}

B

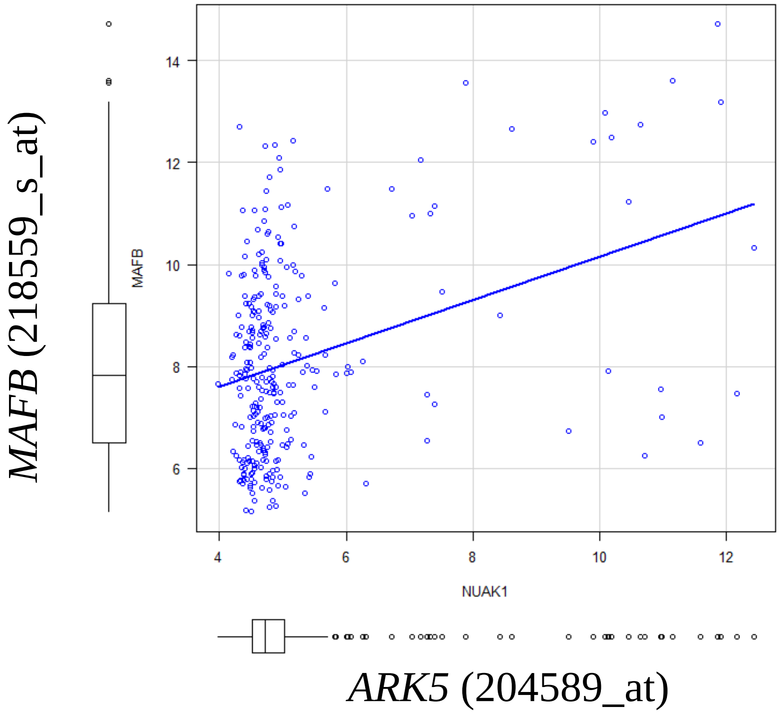

Spearman’s rank correlation coefficient (n = 290)

R = 0.214 P = 2.4 $\times$ 10^{-4}

Figure legends S4:Correlation coefficient analyses of ARK5 and MAF genes in patients with MM. The expression values of ARK5, MAF, and MAFB were statistically analyzed using an EZR software. Data were obtained from the GSE19784 data. The correlation coefficient values (R) and probabilities (P) are individually shown. ARK5 and MAF (**A**) and ARK5 and FAFB (**B**).

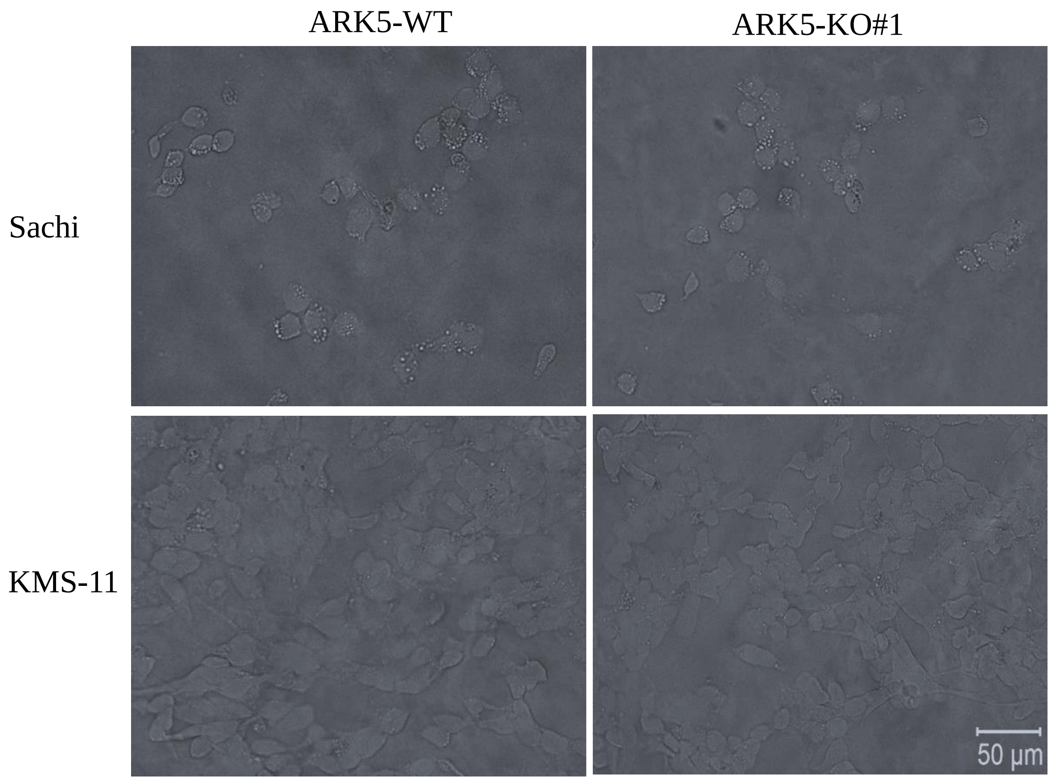

Figure legend S5: Bright field images of the ARK5 WT and ARK5-KO cells.
Representative bright field images showing KMS-11, KMS-11-ARK5-KO#1, Sachi, and Sachi–ARK5-KO#1 in MM cells. The images were received with BZ-II (Keyence) including a fluorescence microscope (BZ-X9000; Keyence)

Supplemental Table S1. Antibodies used in this study

Molecule	Cat number	Company	Species	Dilution
MAF	55013-1AP	Proteintech	Rb	x 2000
MAFB	#30919	CST	Rb	x 2000
Phospho-AKT	#4060	CST	Rb	x 1000
Total-AKT	# 4685	CST	Rb	x 1000
DRP1	AP12957-1	Proteintech	Rb	x 1000
pDRP1	# 4494	CST	Rb	x 1000
MFN1	AP66776-1	Proteintech	Ms	x 1000
MFN2	A19678	ABclonal	Rb	x 1000
ARK5	#4458	CST	Rb	x 2000
OPA1	sc-393296	santa cruz	Ms	x 1000
CDK2	#18048	CST	Rb	x 3000
CDK4	#3136	CST	Ms	x 3000
Caspase9	A2636	ABclonal	Rb	x 3000
Caspase3	#9662	CST	Rb	x 2000
GAPDH	#2118	CST	Rb	x 2000
Anti-rabbit IgG-HRP	#7074	CST	Goat	x 4000
Hoechst 33342	SW150	Dojindo		x 1000
Alexa Fluor Plus 488	A32723	Invitrogen		x 1000
Alexa Fluor® 568	A11011	Invitrogen		x 1000
Mitotracker®	M7512	Invitrogen		x 10000
CST, Cell Signaling Technology; Rb, rabbit; Ms, mouse				

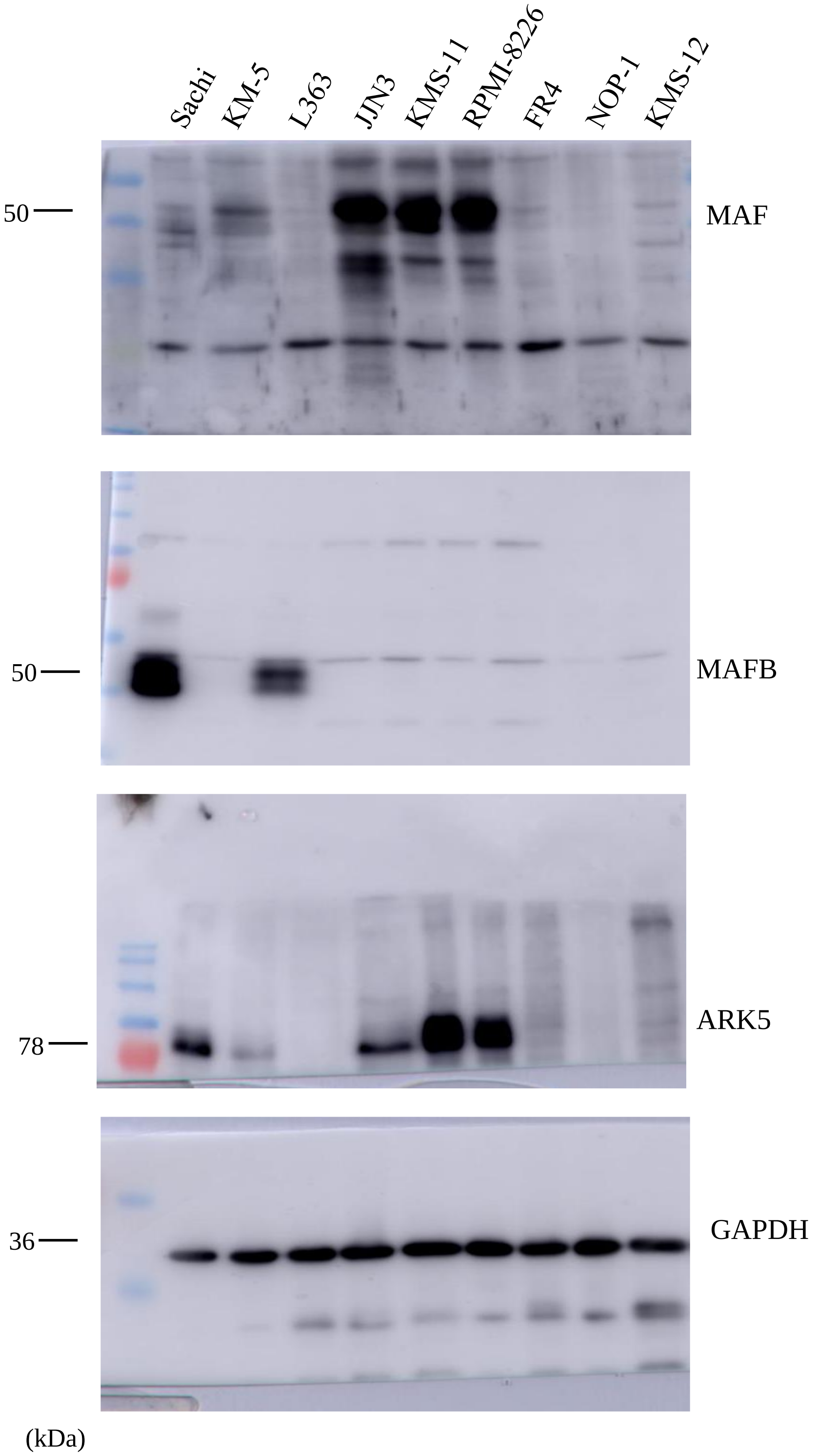

Full and uncropped western blot image of Fig. 1A

Supplemental Fig.S7

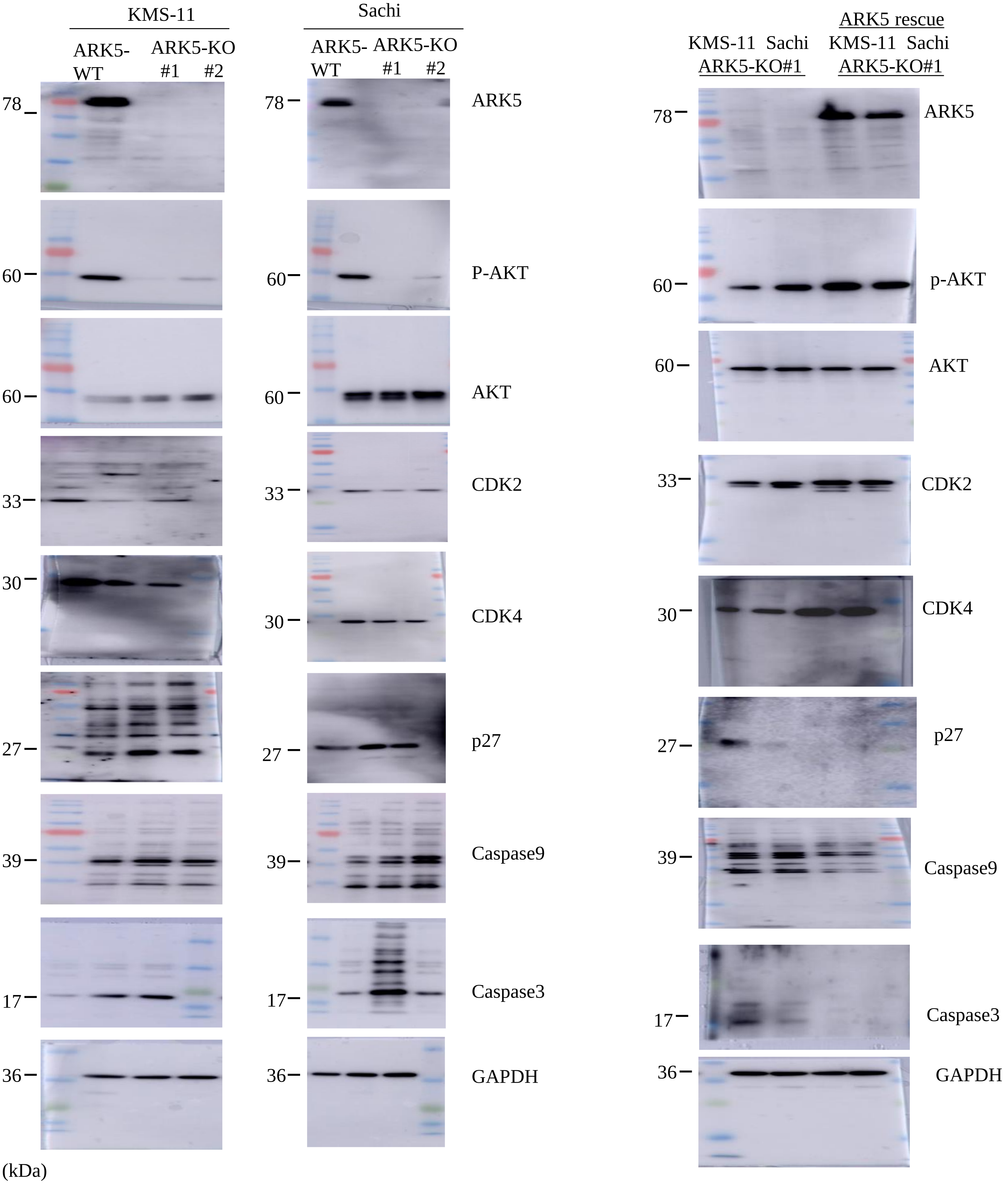

Full and uncropped western blot image of Fig. 3C and D

Supplemental Fig.S8

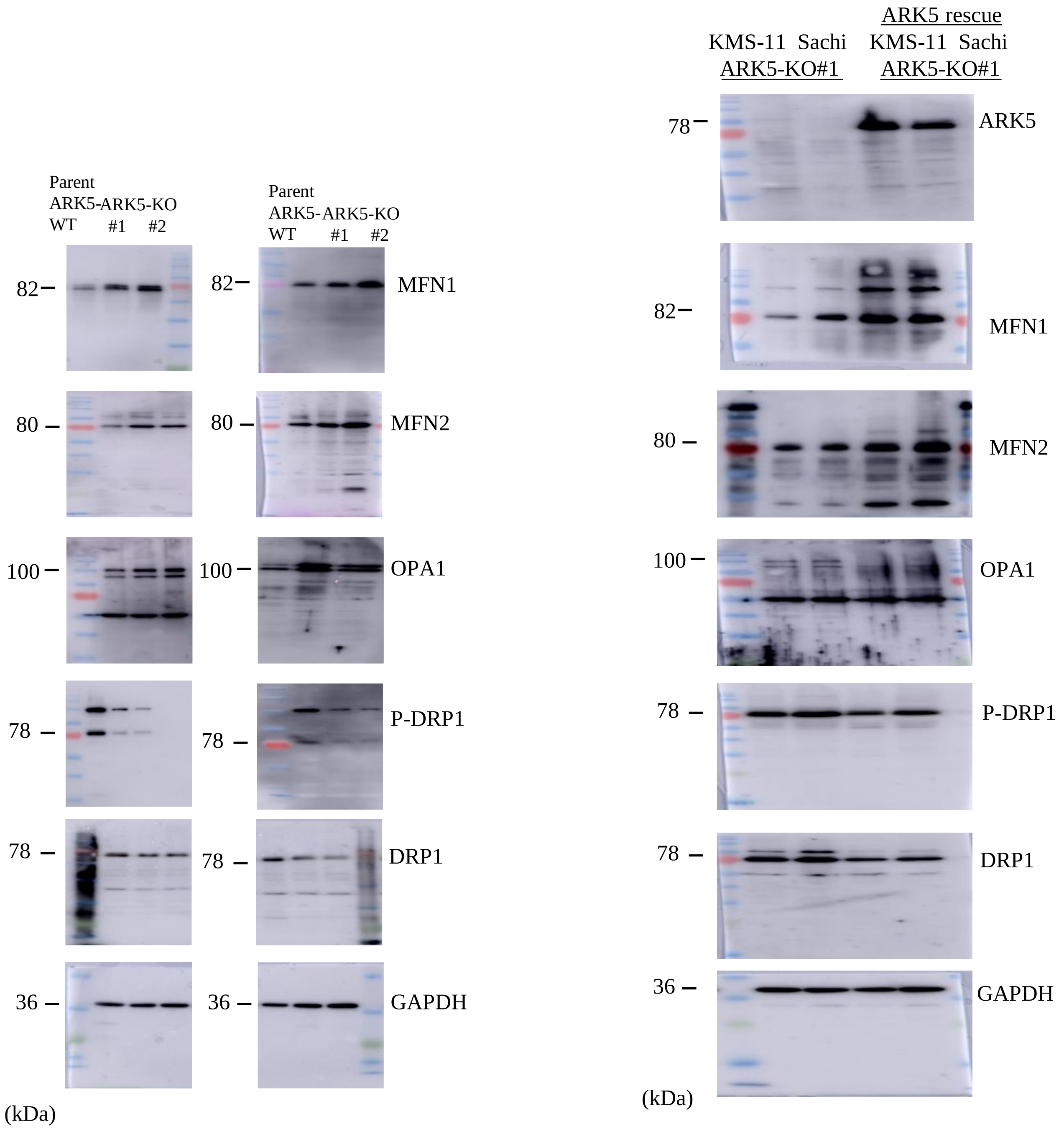

Full and uncropped western blot image of Fig. 5B and C
